# Robust frequency-dependent diffusional kurtosis computation using an efficient direction scheme, axisymmetric modelling, and spatial regularization

**DOI:** 10.1162/imag_a_00055

**Published:** 2024-01-05

**Authors:** Jake Hamilton, Kathy Xu, Nicole Geremia, Vania F. Prado, Marco A.M. Prado, Arthur Brown, Corey A. Baron

**Affiliations:** Centre for Functional and Metabolic Mapping (CFMM), Robarts Research Institute, University of Western Ontario, London, Canada; Department of Medical Biophysics, Schulich School of Medicine and Dentistry, University of Western Ontario, London, Canada; Translational Neuroscience Group, Robarts Research Institute, Schulich School of Medicine and Dentistry, University of Western Ontario, London, Canada; Department of Anatomy & Cell Biology, University of Western Ontario, London, Canada; Department of Physiology and Pharmacology, Schulich School of Medicine and Dentistry, University of Western Ontario, London, Canada

**Keywords:** diffusion MRI, diffusional kurtosis, oscillating gradient, spatial regularization, diffusion dispersion

## Abstract

Frequency-dependent diffusion MRI (dMRI) using oscillating gradient encoding and diffusional kurtosis imaging (DKI) techniques have been shown to provide additional insight into tissue microstructure compared to conventional dMRI. However, a technical challenge when combining these techniques is that the generation of the large b-values (≥2000 s/mm^2^) required for DKI is difficult when using oscillating gradient diffusion encoding. While efficient encoding schemes can enable larger b-values by maximizing multiple gradient channels simultaneously, they do not have sufficient directions to enable the estimation of directional kurtosis parameters. Accordingly, we investigate a DKI fitting algorithm that combines axisymmetric DKI fitting, a prior that enforces the same axis of symmetry for all oscillating gradient frequencies, and spatial regularization, which together enable robust DKI fitting for a 10-direction scheme that offers double the b-value compared to traditional encoding schemes. Using data from mice (oscillating frequencies of 0, 60, and 120 Hz) and humans (0 Hz only), we first show that axisymmetric DKI fitting provides comparable or even slightly improved image quality as compared to kurtosis tensor fitting, and improved DKI map quality when using an efficient encoding scheme with averaging as compared to a traditional scheme with more encoding directions. We also demonstrate that enforcing consistent axes of symmetries across frequencies improves fitting quality, and spatial regularization during fitting preserves spatial features better than using Gaussian filtering prior to fitting, which is an oft-reported pre-processing step for DKI. Thus, the use of an efficient 10-direction scheme combined with the proposed DKI fitting algorithm provides robust maps of frequency-dependent directional kurtosis which may offer increased sensitivity to cytoarchitectural changes that occur at various cellular spatial scales over the course of healthy aging, and due to pathological alterations.

## 1 Introduction

Diffusion MRI (dMRI) is a well-known technique that allows probing of tissue microstructure on spatial scales unattainable with conventional MRI techniques. Diffusional kurtosis imaging (DKI) is a form of dMRI that quantifies the non-Gaussian component of diffusion within tissue, providing greater sensitivity to microstructural changes as compared to conventional diffusion tensor imaging (DTI) (Jensen & Helpern, 2010; Jensen et al., 2005). DKI has been used to study various pathologies such as stroke (Grinberg et al., 2012; Lampinen et al., 2021), mild traumatic brain injury (Stenberg et al., 2021; Stokum et al., 2015), neurodegenerative diseases (Chu et al., 2022; Dong et al., 2020; Falangola et al., 2013; Vanhoutte et al., 2013), as well as many others (Steven et al., 2014), and has shown increased sensitivity to changes in tissue microstructure compared to DTI. An important sequence parameter to consider when interpreting changes in dMRI metrics is the effective diffusion time (Δt_eff_) which determines the length scale within the tissue being probed (Novikov et al., 2014; Tanner & Stejskal, 1968). Commonly used pulsed gradient spin echo (PGSE) sequences to encode diffusion are limited to relatively long Δt_eff_ (>10 ms) for typical pre-clinical gradient hardware (Jelescu et al., 2022; Portnoy et al., 2013), meaning the acquired signal reflects diffusion restriction at multiple spatial scales (i.e., both cellular and subcellular). Conversely, encoding diffusion using an oscillating gradient spin echo (OGSE) sequence allows much shorter Δt_eff_ (corresponding to higher OGSE frequencies), increasing sensitivity to smaller spatial scales (Schachter et al., 2000). By collecting data at multiple gradient oscillation frequencies (i.e., frequency-dependent dMRI), the spatial scale sensitivity can be varied, enabling investigation of tissue microstructure at both subcellular and cellular levels, increasing sensitivity and specificity for studying various pathological conditions. Exploiting the frequency-dependence of DTI metrics has been well-characterized in the healthy brain (Aggarwal et al., 2012; Arbabi et al., 2020; Baron & Beaulieu, 2014; Does et al., 2003), as well as in the study of stroke (Baron et al., 2015), cancer (Iima et al., 2019; Maekawa et al., 2020), and neurodegenerative disease (Aggarwal et al., 2014). Conversely, the dependence of DKI metrics on OGSE frequency is less understood but has been characterized in healthy humans (Borsos et al., 2023; Dai et al., 2023) and rodents (Aggarwal et al., 2020; Pyatigorskaya et al., 2014), as well as in rodent studies of hypoxic-ischemic injury (D. Wu et al., 2018) and a demyelination disease model (Aggarwal et al., 2020).

A key technical challenge in using frequency-dependent DKI is that OGSE sequences are much less efficient at generating the large b-values (≥2000 s/mm^2^) required for DKI compared to PGSE, as b-value is related to the gradient strength (G) and OGSE frequency (f) through $b\sim G^{2}/f^{3}$ (Xu, 2021). Consequently, tetrahedral (four directions) encoding schemes (Conturo et al., 1999) have been required in humans (Borsos et al., 2023) and mice (Aggarwal et al., 2020) to generate the desired b-value for diffusional kurtosis fitting. However, this direction scheme does not allow for directional parameters to be calculated and likely introduces rotational variance in computed metrics (Jones, 2004; Nilsson et al., 2020). Directional (axial and radial) kurtosis parameters have been shown to provide additional specificity to changes in mean kurtosis (Conklin et al., 2016; Goryawala et al., 2018), but estimation of the full kurtosis tensor requires at least two non-zero b-value shells with 15 independent diffusion directions (Lu et al., 2006). A recently developed fitting method, axisymmetric DKI (Hansen et al., 2016), allows accurate fitting of directional kurtosis parameters with two non-zero b-value shells and 9 independent diffusion directions. This method requires determination of a symmetric axis of diffusion within each voxel to which kurtosis parameters are fit to based on the relation between encoding direction and the symmetric axis. Axisymmetric DKI has been shown to generate comparable and even improved kurtosis maps to those computed via kurtosis tensor fitting (Hansen et al., 2016; Nørhøj Jespersen, 2018; Oeschger et al., 2023).

A second challenge when using DKI is noise propagation, primarily due to the high diffusion weighting that is required to accurately compute kurtosis in addition to the large number of parameters to be fit when estimating the kurtosis tensor (i.e., noise amplification) (Glenn et al., 2015; Tabesh et al., 2011). Subsequently, Gaussian smoothing on diffusion-weighted images prior to fitting is widely accepted as a necessary pre-processing step used to reduce noise levels in kurtosis maps (Jensen et al., 2005; Tax et al., 2022). However, because there is averaging of signal from neighboring voxels, this leads to blurring around sharp edges within the image and can cause bias in quantitative analysis (Falconer & Narayana, 1997; Pfefferbaum & Sullivan, 2003; Vos et al., 2011). An alternative approach is to use spatial regularization (Tikhonov et al., 1995) during fitting which balances smoothing of noise with the fitting residual. Regularization has been shown to be advantageous over Gaussian smoothing in functional MRI studies in terms of retaining original image contrast and detection of true areas of activation (Casanova et al., 2009; Liu et al., 2010; Ou & Golland, 2005). It has also been used to control noise amplification in various avenues of dMRI (McGraw et al., 2009; Veraart, Fieremans, et al., 2016; H. Wu & Yan, 2021); however, its use during DKI fitting remains underutilized (Henriques, Jespersen, et al., 2021).

In this study, we aim to delineate an acquisition and analysis scheme capable of generating high-quality frequency-dependent kurtosis maps. We present a 10-direction scheme that is two times more efficient in generating b-value compared to traditional schemes, mitigating a key technical challenge when combining OGSE and DKI techniques. Further, we propose that using data from all frequencies and b-values to estimate the symmetric axis of diffusion will provide a more robust estimate that will reduce noise in parameter maps. We then introduce a two-step spatial regularization algorithm to reduce noise both during symmetric axis determination and kurtosis fitting. We also provide direct comparison demonstrating the advantage of collapsing data from all frequencies to determine the axis of symmetry and using this regularization algorithm over conventional Gaussian smoothing.

## 2 Methods

### 2.1 Human connectome project (HCP) data

Pre-processed dMRI data of a healthy female subject from the HCP1200 release of the Human Connectome Project (HCP) (McNab et al., 2013; Sotiropoulos et al., 2013) was used for validation of axisymmetric DKI fitting and spatial regularization. dMRI data was acquired on a 3 Tesla (T) scanner with parameters: 1.25 x 1.25 x 1.25 mm^3^ voxel size, TE/TR = 89.5/5520 ms. Data consisted of 18 b = 0 images and 3 b-value shells of 1000, 2000, and 3000 s/mm^2^, with 90 encoding directions at each. Data from the highest b-value shell (3000 s/mm^2^) was excluded to ensure the DKI model properly represents the signal decay and to minimize biases from higher order terms not considered in the DKI signal representation which become evident at b-values >2500 s/mm^2^ (Jelescu et al., 2022; Kiselev & Il’yasov, 2007). Full details regarding data acquisition can be found in the HCP 1200 subjects’ reference manual (https://www.humanconnectome.org/study/hcp-young-adult/document/1200-subjects-data-release).

### 2.2 Preclinical mouse data

Data was collected from 8 transgenic mice (4 males) carrying humanized wildtype microtubule-associated protein tau (*mapt*) (Saito et al., 2019) and amyloid precursor protein (*app*) (Baglietto-Vargas et al., 2021) (JAX stock #030898) genes at 6 months of age as part of another ongoing study, and one female mouse of the same genotype and age to compare our efficient direction scheme with a traditional scheme. All animal procedures were approved by the University of Western Ontario Animal Care Committee and were consistent with guidelines established by the Canadian Council on Animal Care. Before scanning sessions, anesthesia was induced by placing mice in an induction chamber with 4% isoflurane and an oxygen flow rate of 1.5 L/min. Throughout the scanning session, isoflurane was maintained at 1.8% with an oxygen flow rate of 1.5 L/min through a custom-built nose cone.

### 2.3 Data acquisition & pre-processing

In vivo MR scanning sessions were performed on a 9.4 T Bruker small animal scanner equipped with a gradient coil insert of 1 T/m strength (slew rate = 4000 T/m/s). Both anatomical and diffusion scans were acquired for each subject. Anatomical images were acquired using a T2-weighted TurboRARE sequence with parameters: in-plane resolution 100 x 100 µm^2^, slice thickness 500 µm, TE/TR = 30/5500 ms, 24 averages, with scan time of 25 minutes. The dMRI protocol included a PGSE sequence (gradient duration = 9.4 ms and diffusion time = 12.4 ms) and OGSE sequences with frequencies of 60 and 120 Hz (corresponding Δt_eff_ of 2.3 and 1.2 ms (Novikov & Kiselev, 2011)). The lowest OGSE frequency (60 Hz) implements the recently introduced frequency tuned bipolar waveforms to reduce the TE of the acquisition (Borsos et al., 2023). The PGSE gradient duration was chosen to fill all available time within the TE determined by the 60 Hz acquisition. For all frequencies, data consisted of 2 b = 0 images and two b-value shells of 1000 and 2500 s/mm^2^ each with the 10-direction scheme outlined in Table 1. This scheme is an efficient 6-direction scheme (Baron & Beaulieu, 2014) combined with a tetrahedral scheme (Aggarwal et al., 2020; Borsos et al., 2023), which offers a factor of two larger b-value than typical schemes due to at least two gradient channels being simultaneously at maximum for each direction. The dMRI protocol was acquired in one integrated scan using single-shot echo planar imaging with 80% of k-space being sampled in the phase encode direction and parameters: in-plane resolution 200 x 200 µm^2^, slice thickness 500 µm, TE/TR = 35.5/15000 ms, 4 averages, total scan time of 66 minutes. To compare the efficient direction scheme with a traditional scheme, two diffusion protocols were acquired during a single scanning session in one mouse. Both protocols had the same scan time of 66 minutes and OGSE frequencies of 0, 60, and 120 Hz. The first protocol was the same as described above: TE/TR = 35.5/15000 ms, 10 directions (Table 1), 4 averages, 4 b = 0 images, and the second (traditional) protocol with parameters: TE/TR = 52/15000 ms, 40 directions isotopically distributed using electrostatic repulsion (Jones et al., 1999), 1 average, 4 b = 0 images. Note the much longer TE required for the second protocol in order to achieve the same b-value with the less efficient direction scheme. For all acquisitions, complex-valued averages were collected as separate repetitions that underwent partial Fourier reconstruction using POCS (Projection onto Convex Sets) (Haacke et al., 1991), phase alignment, frequency and signal drift correction, and Marchenko-Pastur (MP) denoising (Veraart, Novikov, et al., 2016), followed by combining of averages (Rahman et al., 2021). Data then underwent Gibbs ringing correction using MRtrix3 (Tournier et al., 2019) followed by EDDY (Andersson & Sotiropoulos, 2016) from FMRIB Software Library (FSL, Oxford, UK) (Smith et al., 2004) to correct for eddy current-induced distortions. As the partial Fourier encoding nature of the data has been shown to effect pre-processing steps such as MP denoising (Fernandes et al., 2023) and Gibbs ringing correction (Lee et al., 2021), we provide a qualitative and quantitative assessment of their performance in Supplementary Figure 1.

**Table 1.** The 10-direction scheme.

**x**	**y**	**z**
0	1	1
0	1	−1
1	0	1
1	0	−1
1	1	0
1	−1	0
$\sqrt{2/3}$	$\sqrt{2/3}$	$\sqrt{2/3}$
$\sqrt{2/3}$	$\sqrt{2/3}$	$-\sqrt{2/3}$
$\sqrt{2/3}$	$-\sqrt{2/3}$	$\sqrt{2/3}$
$-\sqrt{2/3}$	$\sqrt{2/3}$	$\sqrt{2/3}$

A value of 1 corresponds to the maximum gradient amplitude on a particular channel, which helps illustrate that the gradient norm is increased by $\sqrt{2}$ for the efficient scheme compared to typical direction schemes.

### 2.4 Data fitting

#### 2.4.1 Axisymmetric DKI

The representation of the diffusion-weighted signal in DKI is given by Jensen et al. (2005):

$$\log\left(\frac{S_{b,\hat{n}}}{S_{0}}\right)=bD_{\hat{n}}+\frac{b^{2}}{6}{\bar{D}}^{2}W_{\hat{n}}$$ (1)

where $S_{b,\hat{n}}$ is the diffusion-weighted signal at a particular diffusion weighting, b, and encoding direction $\hat{n}$, $S_{0}$ is the signal intensity at b= 0, $D_{\hat{n}}$ is the diffusion tensor, and $W_{\hat{n}}$ is the diffusional kurtosis tensor measured along $\hat{n}$. As outlined by Hansen et al. (2016), in systems assumed to have axial symmetry (i.e., in the diffusion tensor, $\lambda_{2}=\lambda_{3}$), the kurtosis tensor is characterized by three independent parameters: mean kurtosis tensor ($\bar{W}$), radial tensor kurtosis ($W_{⊥}$), and axial tensor kurtosis ($W_{\left|\right|}$). In such a system, the elements of W and D measured along any diffusion direction are given by:

$$W(\theta )=\frac{1}{16}\left(\begin{array}{c} \cos(40)(10W_{⊥}+5W_{\left|\right|}-15\bar{W}\\ +8\cos(20)(W_{\left|\right|}-W_{⊥})-2W_{⊥}+3W_{\left|\right|}+15\bar{W} \end{array}\right)$$ (2)

and

$$\begin{array}{c} D\left(\theta \right)=D_{⊥}+\cos^{2}\left(\theta \right)\left(D_{∥}-D_{⊥}\right) \end{array}$$ (3)

where θ is the polar angle between the axis of symmetry (which is kept fixed throughout the fitting process) and the diffusion encoding direction, $D_{⊥}$ is the radial diffusivity, and $D_{∥}$ is the axial diffusivity. Subsequently, mean diffusivity ($\bar{D}$), fractional anisotropy (FA), axial kurtosis ($K_{∥}$), and radial kurtosis ($K_{⊥}$) can be calculated as:

$$\begin{array}{l} \bar{D}=\frac{2D_{⊥}}{3}+\frac{D_{\left|\right|}}{3}\;\;\;FA=\sqrt{\frac{3}{2}\frac{{\left(D_{\left|\right|}-\bar{D}\right)}^{2}+2{\left(D_{⊥}-\bar{D}\right)}^{2}}{D_{\left|\right|}^{2}+2D_{⊥}^{2}}}\\ K_{\left|\right|}=\frac{W_{\left|\right|}^{2}\cdot {\bar{D}}^{2}}{D_{\left|\right|}^{2}}\;\;\;\;\;\;\;K_{⊥}=\frac{W_{⊥}^{2}\cdot {\bar{D}}^{2}}{D_{⊥}^{2}} \end{array}$$ (4,5,6,7)

It is important to note that the mean and radial kurtosis quantities calculated here are distinct from conventional mean and radial kurtosis quantities reported from DKI (Henriques, Correia, et al., 2021; Tabesh et al., 2011), as we use the definitions proposed in Hansen et al. (2016).

#### 2.4.2 Spatial regularization

For maps with spatial regularization, a two-step algorithm was used to: (1) provide a robust estimate of the symmetric axis in each voxel, and (2) reduce noise amplification in parameter maps during the fitting process. Regularization was implemented using the conjugate gradient method (Ginsburg, 1963), solved with ordinary least squares optimization. Ordinary least squares was chosen for all optimizations as this method is known to have reduced bias compared to weighted least squares (Morez et al., 2023).

*Step One:* In the first step, fitting of the diffusion tensor was regularized for the purpose of determining the axis of symmetry within each voxel, using isotropic total variation (Rudin et al., 1992), where $\gamma_{DT}$ controls the strength of regularization:

$$\arg\min(||A_{DTI}X_{DT}-y|{|}_{2}^{2}+\gamma DT||T_{DT}X_{DT}|{|}_{2}^{2})$$ (8)

The data consistency term is based on the diffusion tensor $x_{DT}$, the encoding matrix for the diffusion tensor representation $A_{DTI}$, and the log-transformed signal data y. The n’th row of $A_{DTI}$ is given by:

$$A_{DTI,n}=\left(1,-b_{xx,n},-b_{yy,n},-b_{zz,n},-2b_{xy,n},-2b_{xz,n},-2b_{yz,n}\right)$$ (9)

where n counts the diffusion directions acquired, and for the m’th voxel, $x_{DT,m}=(\log\left(S_{0,m}\right),D_{xx,m},D_{yy,m},D_{zz,m},D_{xy,m},$$D_{xz,m},D_{yz,m})$, where $D_{ij}$ are components of the symmetric diffusion tensor (i.e., $x_{DT}$ is a vector of length 7N, where N is the total number of voxels). For each voxel position m, the operator $T_{DT,m}$ performs a numerical derivative, $d_{i}$, along each of the three spatial dimensions, for each diffusion tensor component:

$$\begin{array}{c} T_{DT,m}x_{DT,m}=\left(\begin{array}{l} w_{xx}d_{x}D_{xx,m},w_{xx}d_{y}D_{xx,m},w_{xx}d_{z}D_{xx,m},\\ w_{yy}d_{x}D_{yy,m},\ldots ,w_{yz}d_{z}D_{yz,m} \end{array}\right) \end{array}$$ (10)

where $w_{ij}$ is a constant that scales results. For all cases here, $w_{xx}=w_{yy}=w_{zz}=1$ and $w_{xy}=w_{xz}=w_{yz}=2$ to account for diffusion tensor cross-terms typically having smaller magnitudes than the diagonal of the tensor. Importantly, having the different directions treated equally to each other in $w_{ij}$ for both the diagonal and cross-terms of the diffusion tensor, as was done here, maintains rotational invariance. The total size of $T_{DT}x_{DT}$ is 18N, which corresponds to three derivatives for each of the six unique elements of the diffusion tensor.

*Step Two:* In the second step, we regularize the axisymmetric DKI fitting for the purpose of controlling noise amplification in parameter maps using:

$$\arg\min(||A_{DKI}X_{DK}-y|{|}_{2}^{2}+\gamma_{DK}||T_{DK}X_{DK}|{|}_{2}^{2})$$ (11)

where $x_{DK}$ are the diffusional kurtosis parameters, $A_{DKI}$ is the encoding matrix for the axisymmetric DKI model, and $\gamma_{DK}$ is the regularization weighting for this step. The n’th row of $A_{DKI}$ is given by (Eq. 2):

$$\left(\begin{array}{l} 1,-b_{n}(1-\cos^{2}\theta_{n}),-b_{n}(\cos^{2}\theta_{n}),\\ \frac{b_{n}^{2}}{6}(\frac{10}{16}\cos(4\theta_{n})-\frac{8}{16}\cos(2\theta_{n})-\frac{2}{16},\\ \frac{b_{n}^{2}}{6}(\frac{5}{16}\cos(4\theta_{n})+\frac{8}{16}\cos(2\theta_{n})+\frac{3}{16},\\ \frac{b_{n}^{2}}{6}(\frac{5}{16}\cos(4\theta_{n})+\frac{15}{16}) \end{array}\right)$$ (12)

where $\theta_{n}$ is determined from the dot product of the diffusion encoding vector with the symmetric axis of diffusion within each voxel (principal eigenvector of the fitted diffusion tensor from step one, which is kept fixed throughout fitting), and for the m’th voxel, $x_{DK,m}=(\log\left(S_{0,m}\right),D_{⊥,m},$$D_{∥,m},{\bar{D}}^{2}W_{⊥,m},{\bar{D}}^{2}W_{∥,m},{\bar{D}}^{2}{\bar{W}}_{m})$. Similar to step one, the operator $T_{DK,m}$ performs a numerical derivative in every voxel along each of the three spatial dimensions, for each diffusion parameter in $x_{DK,m}$:

$$\begin{array}{c} T_{DK,m}x_{DK,m}=\left(d_{x}D_{⊥,m},d_{y}D_{⊥,m},d_{z}D_{⊥,m},d_{x}D_{∥,m},\ldots ,d_{z}{\bar{D}}^{2}{\bar{W}}_{m}\right) \end{array}$$ (13)

which has a total size of 15N. Units of ms/µm^2^ are used for b-values in all calculations to ensure that all the entries of $T_{DK,m}x_{DK,m}$ are of comparable magnitude to each other since typically b ~ 1 ms/µm^2^. A “base regularization” (mouse data: $\gamma_{DT}=0.5,\;\;\gamma_{DK}=0.075$, human data: $\gamma_{DT}=0.5,\;\;\gamma_{DK}=0.2$) was heuristically chosen such that regularization at both steps contributed to the reduction of noise in subsequent parameter maps while avoiding over-regularization. To investigate the effect of varying the net regularization, the base regularization weightings were multiplied by a single factor.

Our implementation of axisymmetric fitting with optional spatial regularization is available in the MatMRI toolbox at https://gitlab.com/cfmm/matlab/matmri (Baron, 2021; Varela-Mattatall et al., 2023).

### 2.5 Data analysis

#### 2.5.1 Comparing kurtosis tensor vs. axisymmetric DKI fitting

The DIPY project (Henriques, Correia, et al., 2021) was used to compute kurtosis metrics via kurtosis tensor fitting to compare with the axisymmetric fitting method. Ordinary least squares was used for fitting the full kurtosis tensor (Tabesh et al., 2011). To provide accurate comparison between fitting methods, radial, axial, and mean kurtosis for the full kurtosis tensor was computed based on the definitions proposed by Hansen et al. (2016). While mathematically equivalent mean and axial kurtosis estimates can be directly extracted from the axial kurtosis and mean kurtosis tensor metrics computed in DIPY (Henriques, Correia, et al., 2021), radial kurtosis as defined by Hansen et al. (2016) was computed using implementations available in https://github.com/RafaelNH/Radial_Tensor_Kurtosis. As the principal eigenvector from the diffusion tensor computed from DKI fitting is used as a reference for fitting the kurtosis tensor parameters for DIPY, we used this same method to determine the symmetric axis for axisymmetric DKI fitting where applicable (30- and 90-direction data in humans, and 40-direction data in mice) for these comparisons. For 10-direction data in both humans and mice, we used only the low (1000 s/mm^2^) b-value shell to compute the axis of symmetry, as this showed similar results to when calculated using the diffusion tensor from DKI fitting. When reducing the number of diffusion directions for both fitting methods in HCP data, the reduced direction set that was most uniformly distributed along a sphere based on electrostatic repulsion was selected (Jones et al., 1999). It should be noted that the 10 directions used for the HCP data are not the same as the scheme outlined in Table 1. All b = 0 images were used when the number of directions was reduced. No spatial regularization was performed for these comparisons.

#### 2.5.2 Comparing image quality between efficient and traditional direction schemes

To provide a quantitative comparison of data acquired using the efficient encoding scheme with averaging (10 directions, 4 averages) and a traditional scheme (40 directions, no averaging), we calculated the signal-to-noise ratio (SNR) of b = 0 images from both schemes as the voxel-wise signal mean divided by the voxel-wise signal standard deviation across b = 0 acquisitions. The mean SNR was computed within a region-of-interest (ROI) placed in the cortex.

#### 2.5.3 Variation of principal eigenvector with frequency

To determine the validity of using data from all OGSE frequencies to fit the diffusion tensor for the purpose of finding the symmetric diffusion axis, variation of the principal eigenvector with frequency was examined. A mask was manually drawn around the brain and the central angle between the three-dimensional principal eigenvectors was found via the great-circle distance between them.

#### 2.5.4 Methods to calculate the symmetric axis of diffusion

We hypothesize that the symmetric axis of diffusion depends negligibly on b-value and OGSE frequency and, accordingly, it would be most robust to include data from all frequencies and b-values together when computing the axis of symmetry. To test this hypothesis, maps were computed with three different ways of determining the symmetric axis: (1) AFAB—Using data from all frequencies and all b-values to compute a single diffusion tensor in each voxel, with data from each frequency using the same symmetric axis for parameter fitting. (2) SFAB—Using data from all b-values to compute a diffusion tensor in each voxel for each frequency separately, with parameter fitting for each frequency using a different symmetric axis within each voxel (i.e., the entire fitting process is done independently for each frequency). (3) SFLB—Using data from only the low b-value shell (1000 s/mm^2^) to compute a diffusion tensor in each voxel for each frequency separately, with subsequent parameter fitting for each frequency using data from all b-values.

To quantitatively compare these approaches, we calculated the contrast-to-standard-deviation ratio (CSR) (Kingsley & Monahan, 2005) in manually defined white (WM) and grey matter (GM) regions (Supplementary Fig. 2) as:

$$CSR_{K⊥}=\left|\frac{K_{⊥WM}-K_{⊥GM}}{\sigma_{K_{⊥}-WM+\sigma_{K_{⊥}}^{2}-GM}^{2}}\right|$$ (14)

where $K_{{⊥}_{WM/GM}}$ is the mean $K_{⊥}$ value and $\sigma_{K_{⊥}-WM/GM}$ is the standard deviation of $K_{⊥}$ in the white/grey matter ROIs. All reported values are the mean over all subjects (n = 8 for mice and n = 1 for human data). To provide a measure of noise levels in diffusion and kurtosis parameter maps, we report the mean and standard deviation within ROIs. For HCP data, we used the manually defined WM (corpus callosum) and GM (thalamus) regions in Supplementary Figure 2. For mice, ROI masks of the hippocampus and corpus callosum were generated from the Turone Mouse Brain Atlas (Klein et al., 2009). First, the atlas was registered to a single “chosen T2” volume from one of the scanning sessions using affine and symmetric diffeomorphic transforms with ANTs software (Avants et al., 2011). Similarly, the “chosen T2” volume was registered into each subject’s dMRI native space. The outputted deformation fields and affine transforms were then used to bring both ROIs into the native space of each subject. Masks were visually inspected to ensure good registration quality.

#### 2.5.5 Assessing bias when implementing spatial regularization

To assess any potential bias being introduced by our spatial regularization algorithm, we compared how increasing spatial regularization on 10-direction data compares with “ground truth” (90-direction data) diffusivity and kurtosis parameters in representative WM and GM ROIs in human data (Supplementary Fig. 2).

#### 2.5.6 Comparing spatial regularization vs. Gaussian smoothing

To compare noise levels in kurtosis maps when using Gaussian smoothing and our spatial regularization algorithm, we chose to use $K_{⊥}$ maps as they showed the best contrast between WM and GM. We implemented our regularization algorithm (during fitting) and Gaussian smoothing (prior to fitting) on diffusion-weighted images at various strengths which were chosen so that at each level both maps had an approximately equal visual appearance of noisy voxels. Comparisons between both methods were assessed qualitatively and quantitatively using CSR calculations as outlined in Eq. 14 and their effect on population mean and inter-subject variability was assessed using the Atlas-defined registration procedure outlined above. We performed a two-way ANOVA for each metric to determine if regularization and/or Gaussian smoothing had a significant effect on population diffusivity and kurtosis values across frequencies.

## 3 Results

### 3.1 Kurtosis tensor vs. axisymmetric DKI fitting

**Fig. 1.**
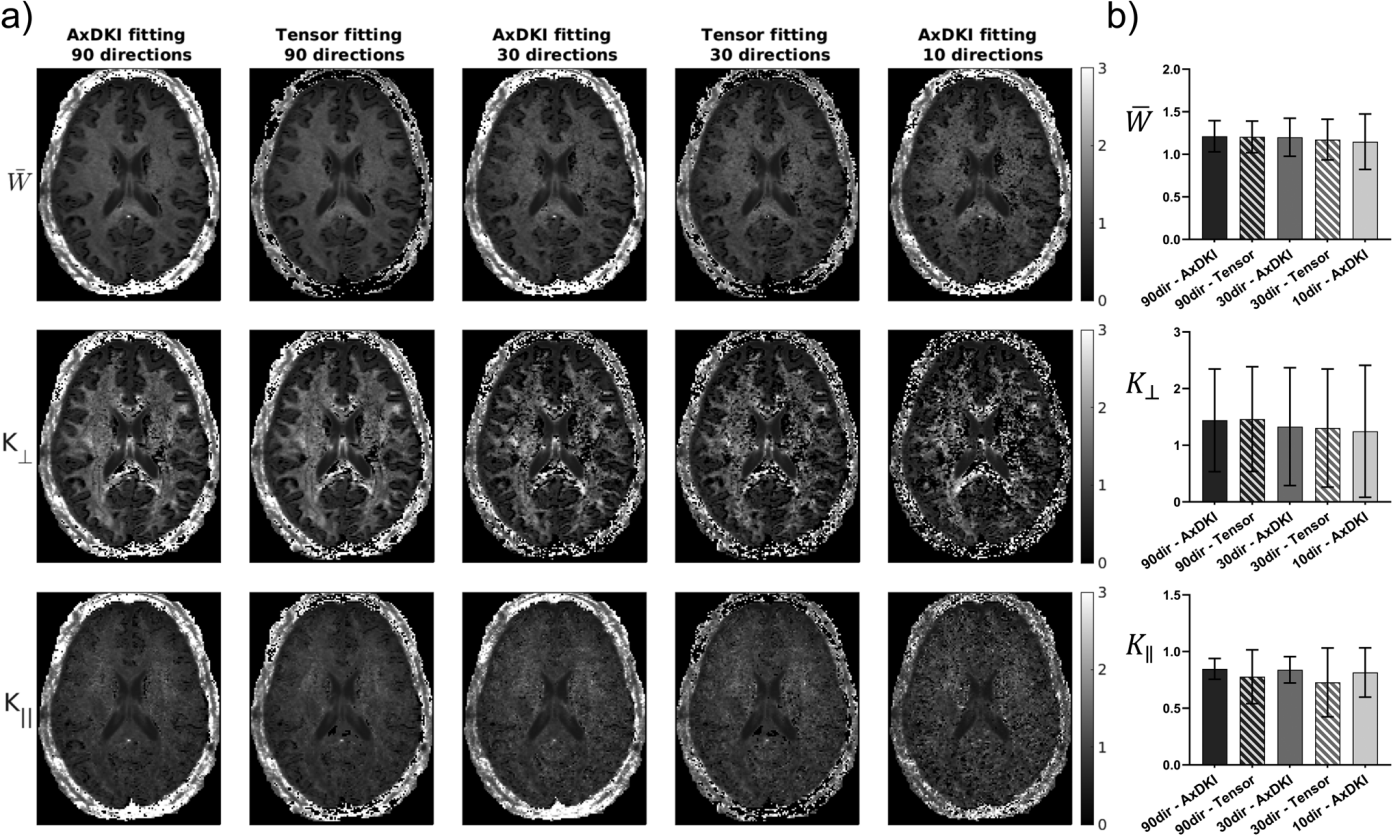
Comparison of kurtosis maps when using conventional kurtosis tensor fitting vs. axisymmetric DKI (AxDKI) fitting at various numbers of diffusion directions. (a) Shows qualitative comparison while (b) shows mean +/- standard deviation of each fitting procedure in the corpus callosum. For AxDKI fitting with 30- and 90-directions, the symmetric axis was estimated with the diffusion tensor calculated from DIPY kurtosis fitting, while for 10-directions it was estimated using data from only the low (1000 s/mm^2^) b-value shell. It should be noted that data shown with 10 directions is not the same scheme as outlined in Table 1.

**Fig. 2.**
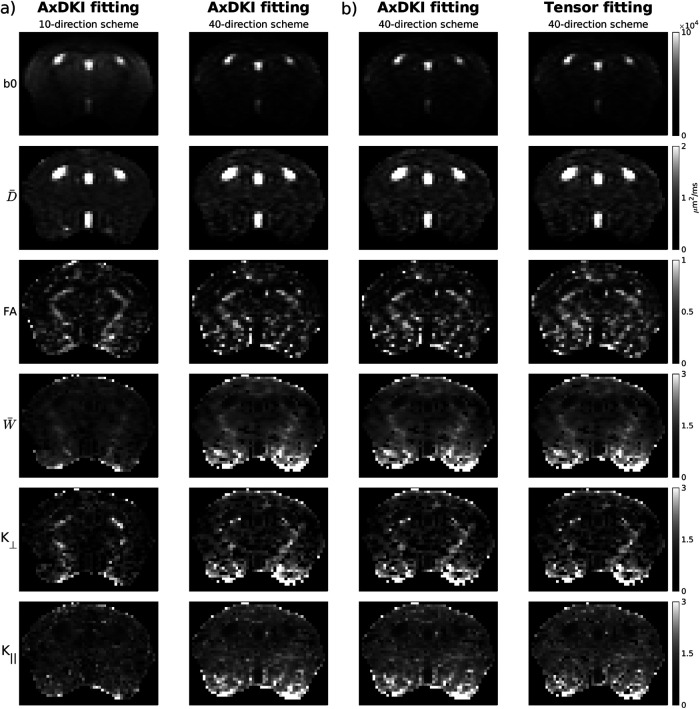
Comparison of b = 0 images, diffusion tensor and kurtosis parameter maps computed with different encoding schemes and fitting methods. (a) Shows comparison between axisymmetric DKI (AxDKI) parameter maps computed with the 10-direction scheme with averaging with a traditional 40-direction scheme, where the symmetric axis for parameter fitting was calculated using the low (1000 s/mm^2^) b-value shell in both cases. (b) Shows comparison between AxDKI and kurtosis tensor fitting with a traditional 40-direction scheme, where the symmetric axis was estimated using the diffusion tensor calculated using DIPY fitting. Data shown is from the PGSE (i.e., 0 Hz) acquisition.

Figure 1 shows human data comparing the two fitting methods at varying numbers of diffusion directions, with no spatial regularization (i.e., $\gamma_{DT}=\;\;\gamma_{DK}=0$). Both methods provide qualitatively similar maps and increased noise levels are seen in kurtosis maps generated from both methods when the number of directions is reduced, as expected. Qualitative differences are difficult to discern when comparing between methods, although slight increases in noise levels can be seen from kurtosis tensor fitting in $\bar{W}$ and $K_{∥}$ maps. Quantitatively, the similarity between methods is evident by near-identical mean and variation of metrics in the corpus callosum, although $K_{∥}$ shows lower mean and higher variation consistent with increased noise levels from kurtosis tensor fitting. These same conclusions are also evident in Figure 2b which shows mouse data comparing the two fitting methods with a traditional 40-direction scheme. Additionally, the efficient 10-direction scheme with averaging results in a much lower TE compared with a traditional 40-direction scheme (35.5 vs. 52 ms), which greatly improved SNR before (16.3 vs. 5.64) and after (30.6 vs. 11.4) pre-processing in b = 0 volumes (Supplementary Fig. 1). Consequently, the 10-direction scheme produces parameter maps with improved contrast and less noise compared to the 40-direction scheme, which show over-estimation of kurtosis parameters and noisy $\bar{D}$ and FA towards the bottom of the brain where there is low SNR (Fig. 2a).

### 3.2 Variation of principal direction of diffusion with OGSE frequency

**Fig. 3.**
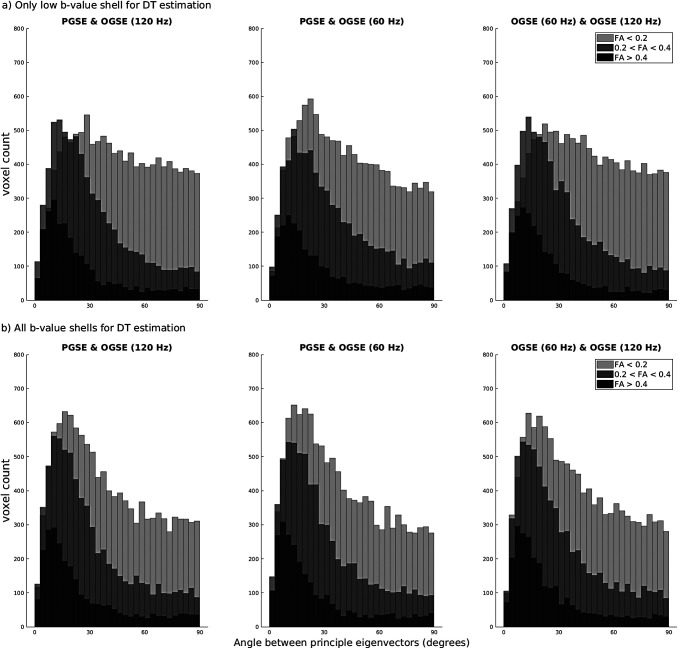
Examining the consistency of principal diffusion direction across PGSE and OGSE frequencies when using various amounts of data for diffusion tensor estimation. (a) Shows histograms which quantify the central angle between principal eigenvectors computed at each frequency when data from only the low (1000 s/mm^2^) b-value shell was used for diffusion tensor estimation, while (b) shows quantification of the central angle between principal eigenvectors computed at each frequency when using data from all b-value shells for diffusion tensor estimation.

Figure 3 shows a histogram of the agreement of the principal eigenvector across examined frequencies when using data from only the low b-value shell (3a) and all b-value shells (3b) for computation. When using only the low b-value shell for diffusion tensor estimation, there is relatively poor agreement of the principal eigenvector across frequencies, especially evident in voxels with low FA values. Comparatively, when using data from all b-value shells, there is much better agreement of the principal eigenvector, especially in voxels with moderate (0.2 < FA < 0.4) and low (FA > 0.4) FA values. Supplementary Figure 3 shows principal diffusion direction maps from each frequency using both methods to calculate the diffusion tensor. Noise-like inconsistencies are evident when using only the low b-value shell to calculate the diffusion tensor which results in discrepancy across frequencies and the histogram (Fig. 3) being more skewed away from collinearity as compared to when using data from all b-value shells.

### 3.3 Comparing methods to calculate the symmetric axis of diffusion

**Fig. 4.**
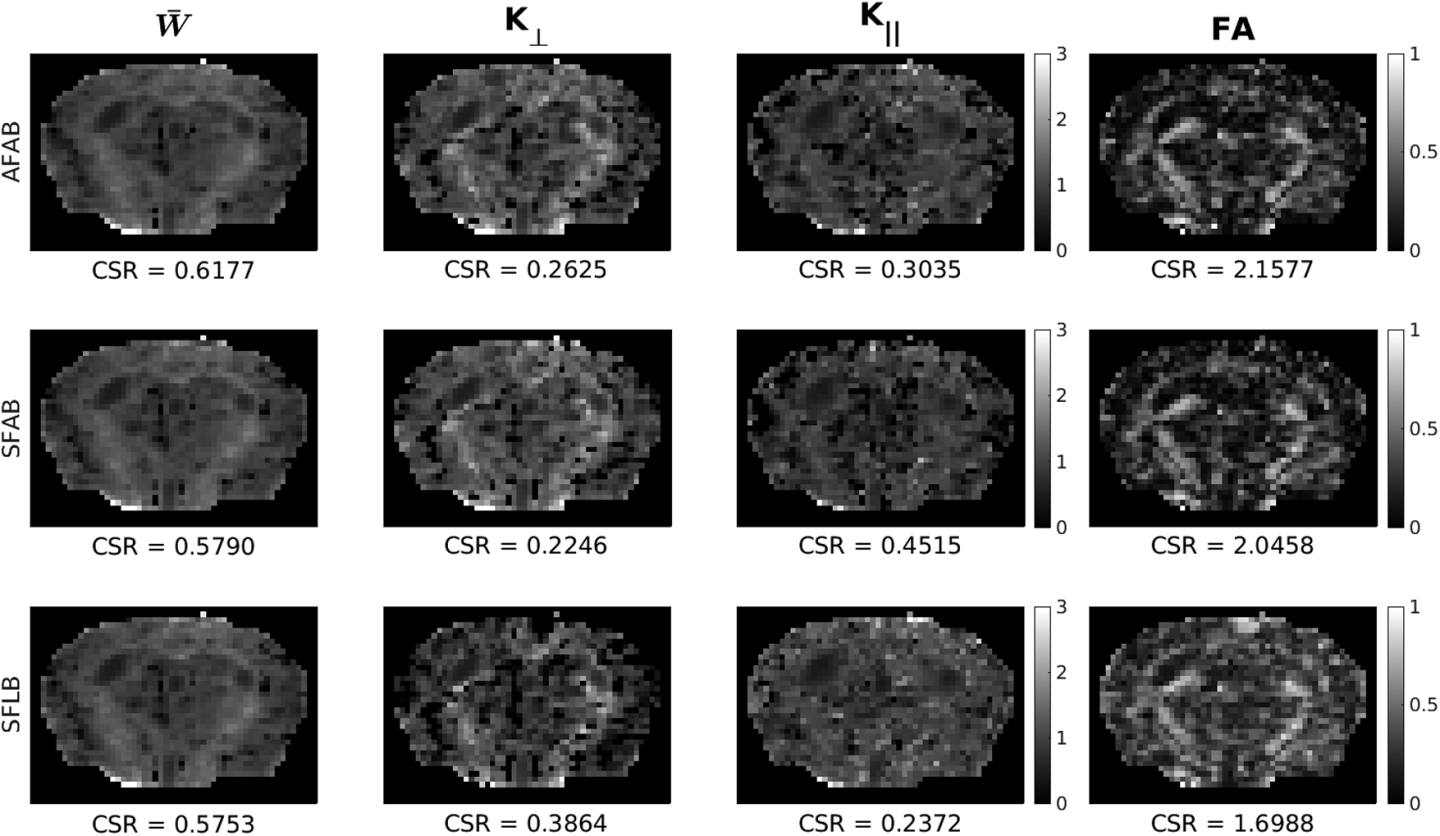
Comparison of kurtosis and FA maps when using various methods to calculate the diffusion tensor for axis of symmetry designation with no regularization (AFAB—using data from all frequencies and b-value shells, SFAB—using data from separate frequencies and all b-value shells, SFLB—using data from separate frequencies and only the low b-value shell). Contrast-to-standard-deviation ratio (CSR) was calculated using Eq. 14 for each subject, and the mean across subjects is reported. Data shown is from the PGSE (i.e., 0 Hz) acquisition.

Figure 4 shows kurtosis and FA maps computed when using differing amounts of data to calculate the symmetric axis of diffusion with no regularization (i.e., $\gamma_{DT}=0$). $\bar{W}$ maps appear qualitatively similar regardless of the method used to calculate the axis of symmetry, with using data from AFAB showing the best CSR. $K_{⊥}$ maps appear the least noisy when using data from AFAB to calculate the axis of symmetry, with quality degradation being most evident when using data from only the low b-value shell (SFLB) to designate the symmetric axis. Although SFLB shows the best CSR for $K_{⊥}$ maps, this is likely due to the increased number of “blackened” voxels in GM. $K_{∥}$ maps show qualitatively minimal white/grey matter contrast no matter the method used to designate the symmetric axis. FA maps also show the best image quality and CSR when using data from AFAB to designate the symmetric axis.

**Fig. 5.**
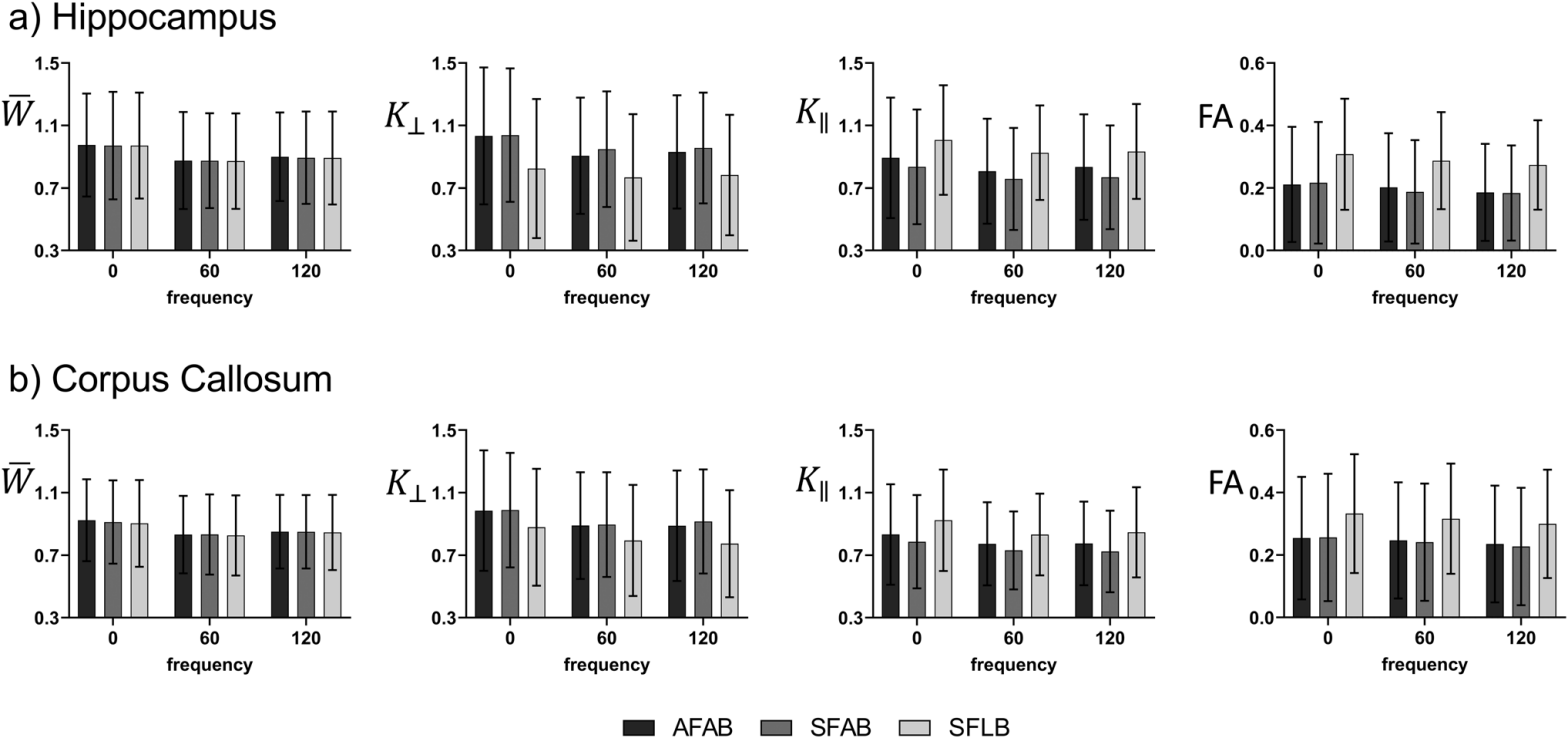
Quantitative data on how collapsing data from all frequencies and using all or only the low b-value shell to compute the axis of symmetry impacts kurtosis and FA values in a representative mouse (AFAB—using data from all frequencies and b-value shells, SFAB—using data from separate frequencies and all b-value shells, SFLB—using data from separate frequencies and only the low b-value shell). (a) Shows data from the hippocampus and (b) shows data from the corpus callosum. All data was measured as mean +/- standard deviation within the ROIs of a single subject.

Figure 5 shows quantitative data when using different methods to calculate the axis of symmetry in the corpus callosum and hippocampus of a single mouse. As seen in Figure 4, $\bar{W}$ appears to be invariant to the axis of symmetry designation. Inclusion of some or all frequencies for diffusion tensor estimation resulted in similar quantitative values. Decreased $K_{⊥}$ and increased FA are seen when using SFLB, consistent with noise contamination. Importantly, the frequency dispersion of all kurtosis metrics remained relatively consistent no matter the method to calculate axis of symmetry. Similar results were observed in other subjects.

### 3.4 Spatial regularization to control noise amplification

**Fig. 6.**
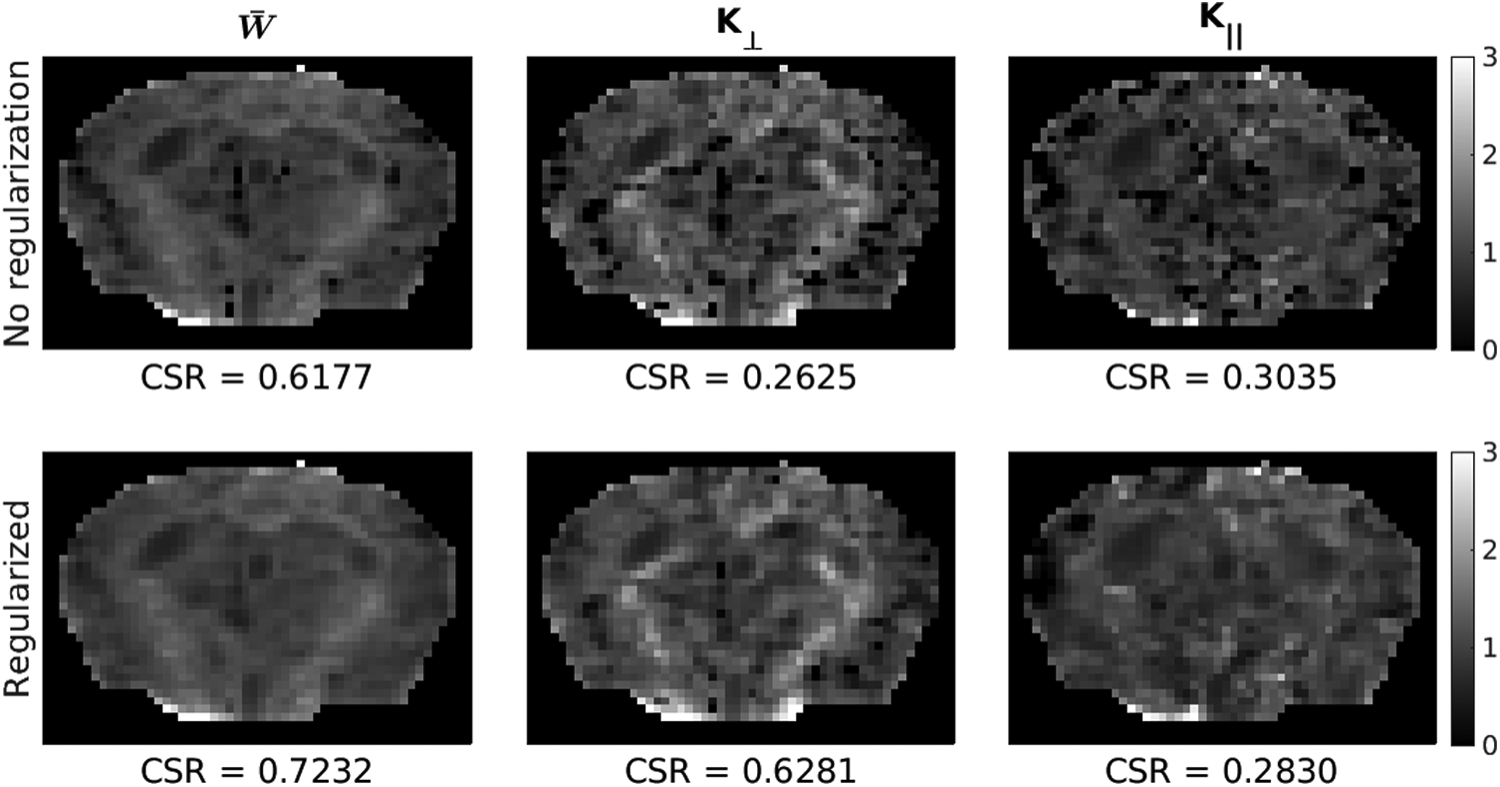
Comparison of kurtosis maps with and without implementation of our two-step regularization algorithm. The top row shows unregularized data and the bottom row shows data that produced high-quality maps while avoiding over-regularization ($\gamma_{\text{DT}}=1.5,\gamma_{\text{DK}}=0.225$). Contrast-to-standard-deviation ratio (CSR) was calculated using Eq. 14 for each subject, and the mean across subjects is reported. Data shown is from the PGSE (i.e., 0 Hz) acquisition.

Figure 6 shows kurtosis maps in mice with and without regularization. Regularization notably reduces the number of noisy voxels in all maps, while there is little-to-no blurring of the original contrast as shown by increases in CSR for $\bar{W}$ and $K_{⊥}$ maps following regularization. This is especially evident in $K_{⊥}$ maps where there is high contrast between WM and GM, which is conserved with regularization.

**Fig. 7.**
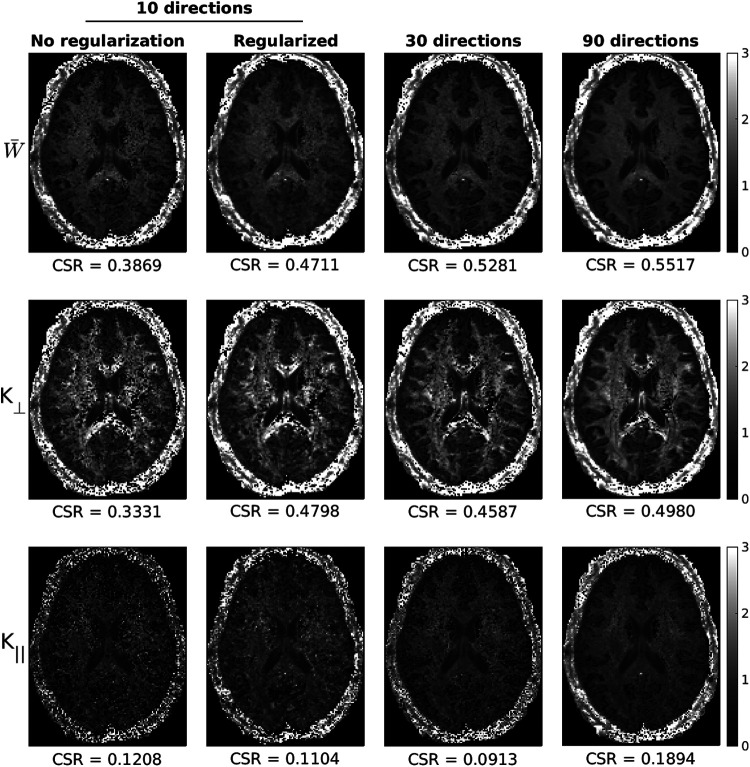
Comparison of kurtosis maps with ($\gamma_{\text{DT}}=1.5,\gamma_{\text{DK}}=0.6$) and without regularization using 10 diffusion directions, and how regularized maps computed with only 10 directions compare with unregularized data computed using 30 and 90 directions. Contrast-to-standard-deviation ratio (CSR) was calculated using Eq. 14 for each map. It should be noted that data shown with 10 directions is not the same scheme as outlined in Table 1.

Figure 7 shows a similar comparison of unregularized and regularized kurtosis maps in human data with only 10 diffusion directions, with unregularized maps computed using 30 and 90 directions shown for comparison. Similar to data shown in mice, regularization greatly reduces the number of noisy voxels in all maps while retaining original contrast as shown by gains in CSR following regularization. It is evident that the regularized data with only 10 directions produce parameter maps with similar or less noise than those acquired with 30 directions, at the expense of slightly reduced contrast at the borders between WM and GM.

**Fig. 8.**
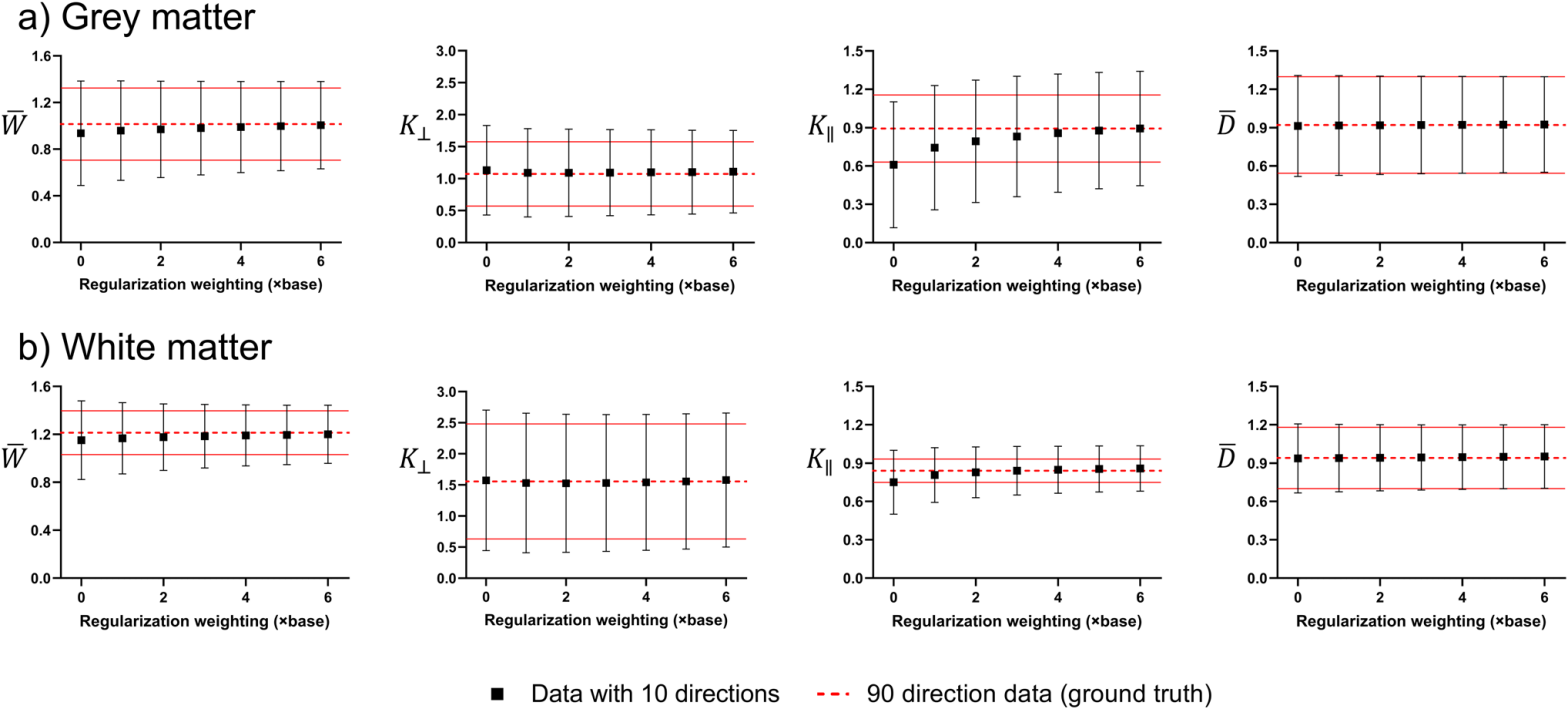
Comparison of 10-direction data with increasing regularization (base: $\gamma_{\text{DT}}=0.5,\;\;\;\gamma_{\text{DK}}=0.2$) and 90-direction (“ground truth”) data in (a) grey and (b) white matter ROIs (Supplementary Fig. 2). All data was measured as mean +/- standard deviation within the same subject. Solid red lines indicate standard deviation of ground truth data.

Figure 8 shows how increasing regularization of 10-direction data compares with 90-direction data (“ground truth”) in human WM and GM ROIs. Quantitatively, we can see that increasing regularization results in negligible bias in kurtosis and diffusivity metrics. $K_{⊥}$ and $\bar{D}$ values remain in agreement with ground truth with increasing regularization, and $\bar{W}$ and $K_{∥}$ values become more aligned with the ground truth following regularization.

**Fig. 9.**
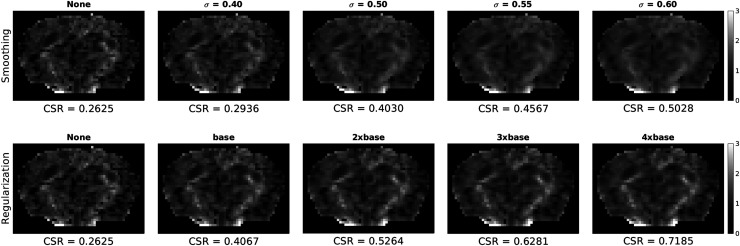
Comparison of ${\text{K}}_{⊥}$ maps with increasing levels of spatial regularization and Gaussian smoothing. σ indicates the standard deviation of the Gaussian kernel used for smoothing on the diffusion-weighted images prior to fitting, in units of voxels. Regularization weighting (base: $\gamma_{\text{DT}}=0.5,\gamma_{\text{DK}}=0.075$) and σ levels were chosen so that in each column there are approximately equal numbers of noisy voxels in each column. Contrast-to-standard-deviation ratio (CSR) was calculated using Eq. 14 for each subject, and the mean across subjects is reported. Data shown is from the PGSE (i.e., 0 Hz) acquisition.

Figure 9 compares $K_{⊥}$ maps from a representative mouse with increasing levels of spatial regularization and Gaussian smoothing. Both methods reduce the number of noisy voxels, however, increasing the level of smoothing blurs the maps such that the contrast between WM and GM becomes less apparent. In comparison, as the regularization weighting is increased, the number of noisy voxels also decreases but the original contrast is preserved. This is evident quantitatively as CSR increased much more with increasing regularization (0.2625 to 0.7185) as compared to Gaussian smoothing (0.2625 to 0.5028). A similar comparison between regularization and smoothing is shown in human data in Supplementary Figure 4, which illustrates the same trends.

**Fig. 10.**
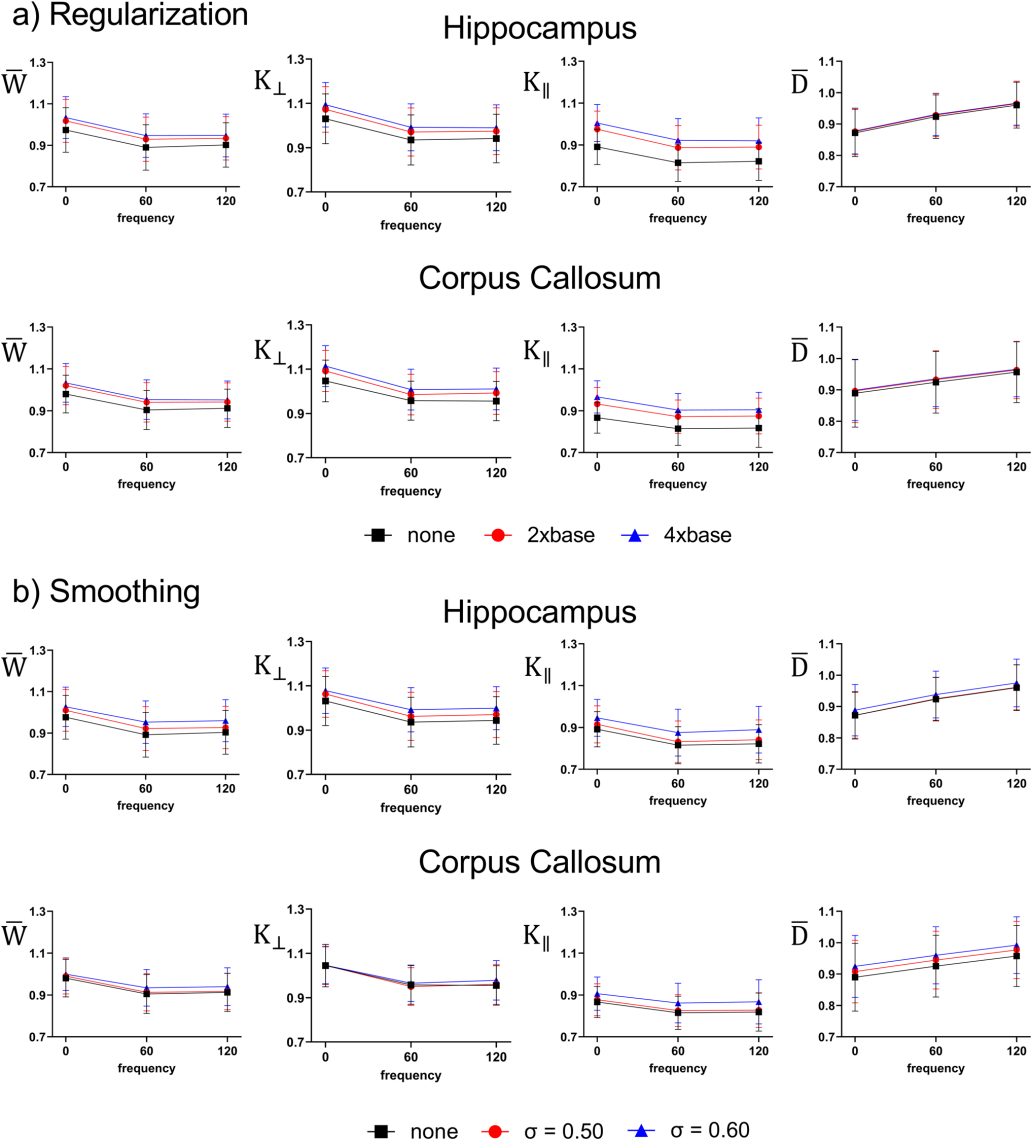
Examining how regularization and Gaussian smoothing impacts the inter-subject variability of kurtosis and diffusivity metrics. (a) Shows how regularization impacts the between-subjects (n = 8) mean and variability within the hippocampus and corpus callosum (base: $\gamma_{\text{DT}}=0.5,\gamma_{\text{DK}}=0.075$). (b) Shows how smoothing impacts the between-subjects mean and variability within each region (σ indicates the standard deviation of the Gaussian kernel used for smoothing on the diffusion-weighted images prior to fitting, in units of voxels). Data shown as mean +/- standard deviation across subjects.

Figure 10 shows how increasing levels of regularization and smoothing impact the inter-subject variability of $\bar{D}$ and kurtosis metrics. It is apparent that with increasing levels of regularization the mean value of kurtosis metrics increases, which is expected as the number of noisy “blackened” voxels decreases. This is evident as we found a significant main effect of regularization strength on $K_{∥}$ values in both the hippocampus (F(2, 67) = 8.190, p < 0.001) and corpus callosum (F(2, 67) = 8.433, p < 0.001), and $K_{∥}$ maps display the largest amount of noise. For Gaussian smoothing there are similar trends, with a larger amount of smoothing increasing the mean values in the hippocampus. However, in the corpus callosum, the mean value for each kurtosis metric remains relatively consistent with increased smoothing while the diffusivity increases (although there is a slight increase in $K_{∥}$ likely due to higher noise levels when regularization is not used). This is likely due to a competing effect whereby noisy voxels are being removed, but there is blurring with nearby cerebrospinal fluid which decreases the kurtosis and increases diffusivity within white matter. No significant changes were found with varying levels of smoothing. This data also depicts that both regularization and Gaussian smoothing have little-to-no effect on the frequency dispersion of diffusivity and kurtosis metrics, and the inter-subject variability remains consistent suggesting this is due to true variation between subjects.

## 4 Discussion

In this study, we investigated a method to compute frequency-dependent directional kurtosis maps, which was used in combination with a 10-direction scheme that has twice the efficiency of traditional schemes in generating b-value. Axisymmetric DKI fitting was shown to provide comparable or even slightly improved kurtosis maps as compared to conventional kurtosis tensor fitting, and the 10-direction scheme with multiple averages was shown to reduce erroneous kurtosis estimation as compared to traditional schemes with a larger number of directions due to the shorter TE enabled by the efficient direction scheme. The principal axis of diffusion did not vary appreciably between the frequencies explored in regions with high FA (i.e., white matter), and we illustrated that using data from all frequencies and b-values for the purpose of determining the symmetric diffusion axis improved parameter map quality. Finally, as kurtosis fitting is susceptible to noise amplification, a two-step spatial regularization algorithm was presented, which reduces noise while preserving contrast and we showed the advantages of using regularization as opposed to conventional Gaussian smoothing for controlling noise amplification in DKI maps.

Axisymmetric DKI fitting and kurtosis tensor fitting were shown to produce qualitatively and quantitatively similar kurtosis maps, with subtle increased noise levels seen from tensor fitting (Figs. 1 and 2) consistent with Hansen et al. (2016). These results agree with two recent simulation studies which found comparable or even slightly improved performance of axisymmetric DKI compared to tensor fitting in terms of parameter estimation (Nørhøj Jespersen, 2018; Oeschger et al., 2023). Nørhøj Jespersen (2018) also showed that axisymmetric DKI generates somewhat more accurate $K_{∥}$ and $K_{⊥}$ estimation compared to tensor fitting in cases of low SNR, which agrees with our observation of differences between methods becoming more apparent when the number of directions was reduced. Our finding of increased noise levels in $K_{∥}$ maps from tensor fitting agrees with Oeschger et al. (2023) who found axisymmetric DKI is particularly advantageous over tensor fitting in terms of $K_{∥}$ estimation. The improved performance of axisymmetric DKI is likely due to the reduced parameter space (8 compared to 22) used compared with tensor fitting, which decreases noise propagation during the fitting process.

The challenge in generating large b-values when using oscillating gradient encoding is evident in both rodent (Aggarwal et al., 2020) and human (Borsos et al., 2023) DKI where tetrahedral diffusion encoding was required to collect data at 140 Hz in mice and 23 Hz in humans. Although slightly less efficient in generating b-value, the 10-direction scheme was shown to generate b-values up to 2500 s/mm^2^ at frequencies up to 120 Hz with typical pre-clinical gradient strengths. While this encoding scheme is not optimized for uniform directional sampling, the trade-off is a large reduction in TE. Data collected with our efficient 10-direction scheme achieved a b-value of 2500 s/mm^2^ at 120 Hz with a TE of 35.5 ms, while when a 40-direction scheme was used it required a TE of 52 ms due to the increased diffusion gradient duration required to achieve the same b-value. Despite the reduced number of encoding directions compared to a traditional DKI scheme with ~30-40 directions (Fukunaga et al., 2013; Tabesh et al., 2011), SNR can be recovered by using signal averaging as was done here. We show in Figure 2 that while scan time is held constant, an efficient encoding scheme with averaging resulted in ~3x higher SNR in b = 0 volumes and produces maps with enhanced contrast in comparison to a traditional direction scheme where there is visible over-estimation of kurtosis metrics in regions with low SNR (bottom of the brain as we use a surface coil placed on the top of the head) due to the increased TE (Zhang et al., 2021). These results complement the findings by Lebel et al. (2012), which showed that a 6-direction protocol with 5 averages gives comparable robustness in DTI metrics as compared to a 30-direction protocol even when the greater b-value efficiency of 6 directions is not exploited and TE’s are held constant.

The most important step in the axisymmetric fitting method is the determination of a symmetric diffusion axis in each voxel, which is acquired by fitting the diffusion tensor and using the principal eigenvector as the symmetric axis. Results showed good agreement of the principal eigenvector across the frequencies explored (0 to 120 Hz), with the best agreement occurring in regions with high FA values and when using all b-values for computation (Fig. 3). Using frequencies of 0 to 150 Hz, Aggarwal et al. (2012) found that the principal diffusion direction in voxels with relatively high FA had no apparent frequency-dependent change. The frequencies explored in this study correspond to hindered diffusion, specifically average molecular displacements of ~2.5-8 µm as determined via the Einstein-Smoluchowski relation (Morozov et al., 2020). As this displacement range is larger than the typical diameters of axons in the mouse brain (<1 µm) (Basu et al., 2023; West et al., 2015), the water molecules will reach the axonal membrane at all frequencies and therefore the principal diffusion direction should not vary between frequencies. This is seen as voxels with high FA values have better agreement between the symmetric axis across frequencies. However, if fibers have high curvature within a single voxel, this would result in true frequency-dependence of the principal diffusion direction via the axial diffusion. Although within-voxel curvature of fiber bundles is likely to be ubiquitous throughout the brain (Cetin Karayumak et al., 2018), we suspect that the resultant variation of the principal diffusion direction with frequency is insignificant compared to the increased robustness to noise that comes from utilizing all frequencies for diffusion tensor estimation in most *in vivo* scenarios. In grey matter which is predominated by neuron, astrocyte, and microglia cell bodies with typical diameters of 5-10 microns (Bushong et al., 2002; Kozlowski & Weimer, 2012; Oberheim et al., 2009), it is possible that the principal diffusion direction could change between the frequencies explored. Nevertheless, in spherically shaped objects, the diffusion tensor eigenvalues are approximately equal and the principal eigenvector is arbitrary and susceptible to noise.

The kurtosis and FA maps presented in Figure 4 show the least amount of noise when using data from all frequencies and b-values to calculate the symmetric axis of diffusion, also shown quantitatively via CSR calculations. This is expected as the principal diffusion direction is unlikely to change with frequency (as outlined above) as well as b-value (Tournier et al., 2013); therefore, using data from all frequencies and b-values will reduce the susceptibility of the diffusion tensor calculation to noise and increase its robustness. The largest gains in map quality were observed when using data from all b-value shells for symmetric axis estimation. It was shown both qualitatively and quantitatively that $\bar{W}$ is invariant to the axis of symmetry designation, whereas $K_{⊥}$ and $K_{∥}$ map quality is dependent on an accurate designation of symmetric axis in agreement with previous findings (Hansen et al., 2016). Importantly, the frequency dispersion of kurtosis metrics remains consistent regardless of the method used to designate the axis of symmetry.

The implementation of the two-step regularization algorithm showed to be advantageous in reducing noise amplification in kurtosis fitting while preserving contrast in both mice and humans evident by increases in CSR following regularization (Figs. 6 and 7). Regularization remains underutilized in dMRI research, despite showing improved image quality in simultaneous multi-slice dMRI (Haldar et al., 2020), compressed sensing (Mani et al., 2021; Varela‐Mattatall et al., 2023), and estimation of fiber orientation distribution functions (McGraw et al., 2009; Sakaie & Lowe, 2007). Our results show that regularization is particularly advantageous in improving parameter map quality in DKI, which are often confounded by noise amplification. Importantly, although this two-step algorithm was presented for the computation of frequency-dependent kurtosis maps, it can also be implemented for data acquired at a single diffusion time.

Axisymmetric DKI fitting has been shown to have comparable or less bias from the ground truth compared to kurtosis tensor fitting (Hansen et al., 2016, 2017; Nørhøj Jespersen, 2018), and we show that our regularization of the fitting does not introduce any additional bias into measured parameters and even produces estimated values closer to the ground truth (Fig. 8). This aligns with a recent study that found regularization of kurtosis tensor fitting reduces the average difference between estimated and ground-truth DKI parameters, increasing the reproducibility of DKI (Henriques, Jespersen, et al., 2021).

While Gaussian smoothing is a widely accepted as a necessary pre-processing step for DKI (Jensen et al., 2005; Tax et al., 2022), a direct comparison between smoothing and the proposed regularization implementation was provided, showing improved map quality and CSR when using regularization in both mice and human data (Fig. 9 and Supplementary Fig. 4). This aligns with functional MRI research where smoothing was shown to lead to a loss of detail caused by blurring of activation regions beyond their true boundaries (Liu et al., 2010). Comparatively, spatial regularization has been shown to improve activity detection and fine details, given that smoothing introduced via regularization is much less severe than Gaussian smoothing (Casanova et al., 2009). When comparing the regularized parameter maps to those obtained with Gaussian smoothing, it is evident qualitatively and quantitatively that regularization preserves image contrast much better than smoothing which causes blurring with adjacent structures and cerebrospinal fluid. Although not explored here, spatial regularization is likely to have advantages over Gaussian smoothing when conducting voxel-wise analyses, as smoothing can lead to large distortions in voxels which vary depending on their neighboring voxels. This is because spatial regularization favors smoothing that does not introduce errors in data consistency (i.e., the $||A_{DKI}X_{DK}-y|{|}_{2}^{2}$ term in Eq. 11), whereas Gaussian smoothing non-specifically smooths all voxels in an image equally. Finally, we showed that both regularization and Gaussian smoothing have little impact on the dispersion of kurtosis metrics with frequency and inter-subject variability, which is of great importance when examining the frequency-dependence of metrics.

This study is not without its limitations. As alluded to previously, based on the sizes of microstructural barriers within tissue, it is not expected that the principal diffusion direction would change across the frequencies explored in this study. However, this assumption of unity of the principal diffusion axis across frequencies may not always be true, such as when using very high OGSE frequencies or in voxels with a high degree of fiber curvature. Additionally, the optimal maximum b-value to be used for *in vivo* DKI fitting remains uncertain, with some suggesting b_max_D~2 (Jensen & Helpern, 2010; Kuo et al., 2018) is sufficient which is lower than the 2500 s/mm^2^ max b-value shell used here. Although the workflow generates high-quality kurtosis maps in mice, further investigation of the proposed method should be investigated in humans. Based on recent work by Dai et al. (2023) who performed frequency-dependent DKI in humans with b-values of 2000 s/mm^2^ and frequencies up to 47.5 Hz with the MAGNUS high performance gradient system, using the more efficient 10-direction scheme and our fitting algorithm could allow for increased map robustness and higher b-values and frequencies to be examined in humans. Additionally, incorporation of spiral trajectories would allow for increased b-value for a given TE as compared to standard Cartesian trajectories (Michael et al., 2022). It is important to note that the isotropic total variation used in our spatial regularization algorithm is not generally rotationally invariant. However, ensuring that all different directions have equal weights ensures rotational variance of Eq. 8 (Eq. 11 is inherently rotationally invariant). Future work may also explore how different types of regularization (i.e., joint regularization (Bredies et al., 2010)) and automatic selection of regularization weighting (Varela‐Mattatall et al., 2021) can be used to control noise amplification more effectively in kurtosis maps. The $\ell_{2}$-norm based regularization method was chosen here because it can be evaluated rapidly using the conjugate gradient method. The computation time was 7 seconds for a full brain mouse dataset and ~1 minute for human HCP (90-direction) dataset on a workstation with a Intel(R) Core(TM) i9-11900K (3.5 GHz) and an NVIDIA GeForce RTX 4090 (24 GB). While the performance of the pipeline is much better with a GPU, it is feasible on CPU (computation times of 1.5 and 75 minutes for mouse and human datasets, respectively).

## 5 Conclusions

In conclusion, we presented a workflow to generate robust frequency-dependent kurtosis maps in mice. The 10-direction encoding scheme presented is twice as efficient in generating b-value compared to traditional schemes, and axisymmetric DKI fitting was shown to provide comparable or even improved kurtosis maps compared to conventional tensor fitting and has reduced dataset requirements that enables fitting with a 10-direction scheme. We showed that using this 10-direction scheme with multiple averages is advantageous in terms of kurtosis parameter estimation as opposed to traditional schemes with ~30-40 encoding directions. Furthermore, taking advantage of degeneracies across frequencies and implementing a two-step regularization algorithm was shown to decrease noise amplification while preserving image contrast. While the interpretation of changes in frequency-dependent kurtosis parameters remains unclear, this workflow will allow further study of how these changes relate to tissue microstructure. Specifically, the advances presented here have the potential to allow for increased sensitivity to cytoarchitectural changes at various spatial scales (i.e., subcellular and multicellular organizational levels) over the course of aging and in various pathological conditions.

## Data and Code Availability Statement

Code for our implementation of axisymmetric DKI fitting with optional spatial regularization is available at https://gitlab.com/cfmm/matlab/matmri. Human data used in this study are available from the Human Connectome Project at https://www.humanconnectome.org/study/hcp-young-adult/document/1200-subjects-data-release, and mouse data are available upon reasonable request.

## Author Contributions

Jake Hamilton: Conceptualization, Data curation, Formal analysis, Investigation, Visualization, Writing—original draft, and Writing—review & editing. Kathy Xu: Project administration, Resources, and Writing—review & editing. Nicole Geremia: Project administration, Resources. Vania F. Prado: Methodology, Resources. Marco A.M. Prado: Methodology, Resources. Arthur Brown: Funding acquisition, Project administration, Resources, Supervision, and Writing—review & editing. Corey A. Baron: Conceptualization, Funding acquisition, Resources, Software, Supervision, and Writing—review & editing.

## Declaration of Competing Interest

The authors declare no competing interests.

## Supplementary Material

Supplementary Material
