# Supplementary material for: Robust frequency-dependent diffusional kurtosis computation using an efficient direction scheme, axisymmetric modelling, and spatial regularization
Source: Imaging Neurosci (Camb). 2024 Jan 5;2:imag-2-00055. doi: 10.1162/imag_a_00055 (PMC12224416; doi:10.1162/imag_a_00055)
Supplement: Supplementary Material [file imag_a_00055-supp.pdf]

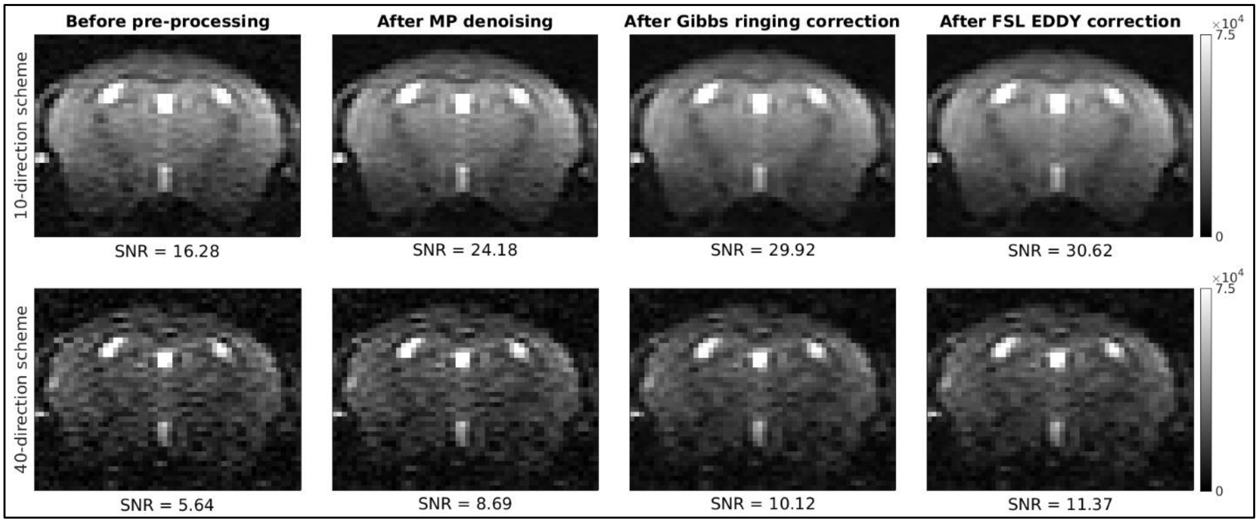

Supplementary Figure 1. *The effect of pre-processing (Marchenko-Pastur (MP) denoising, Gibbs ringing correction, and eddy current correction) on b=0 volumes from two acquisition schemes (Top – 10 encoding directions (Table 1), 4 averages, TE = 35.5 ms, Bottom – 40 encoding directions, 1 average, TE = 52 ms). Signal-to-noise ratio (SNR) of b=0 images from both schemes was calculated as the voxel-wise signal mean divided by the voxel-wise signal standard deviation across b=0 acquisitions. The mean SNR was computed within a region-of-interest (ROI) placed in the cortex.*

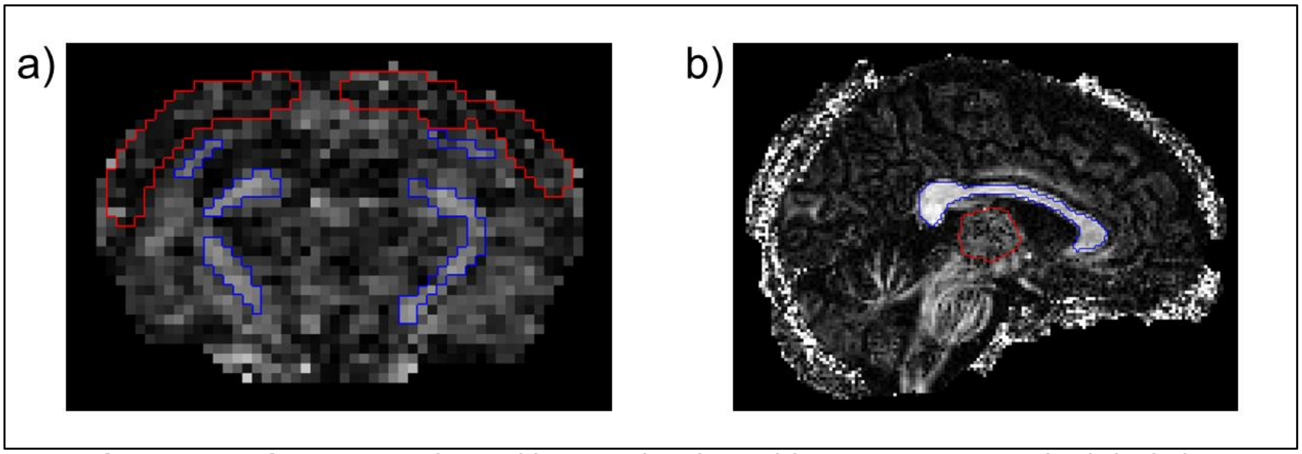

Supplementary Figure 2. *Regions-of-interest (ROI) used for contrast-to-standard-deviation ratio (CSR) calculations. a) shows chosen mouse WM and GM ROIs, b) shows chosen human WM (corpus callosum) and GM (thalamus) ROIs. All ROIs were drawn on multiple slices, single slices shown for illustrative purposes. Blue outline indicates the white matter ROI and red outline indicates the grey matter ROI.*

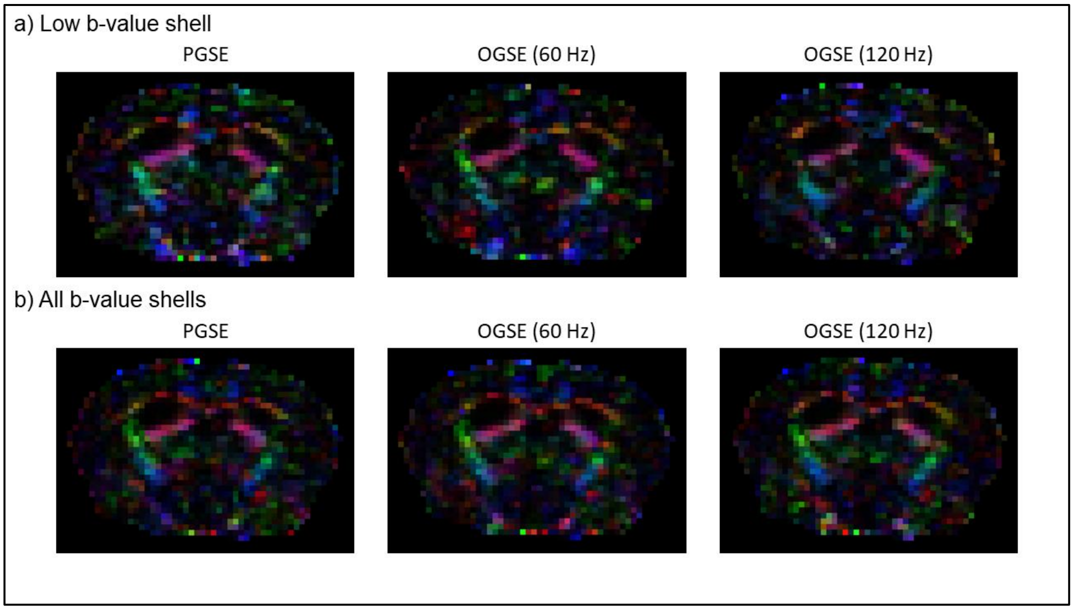

Supplementary Figure 3. *Principal diffusion direction maps when the diffusion tensor is calculated using a) only the low (1000 s/mm^2) b-value shell, and b) all b-value shells. FA maps generated from using only the low b-value shell were used to weigh all maps to provide an accurate comparison.*

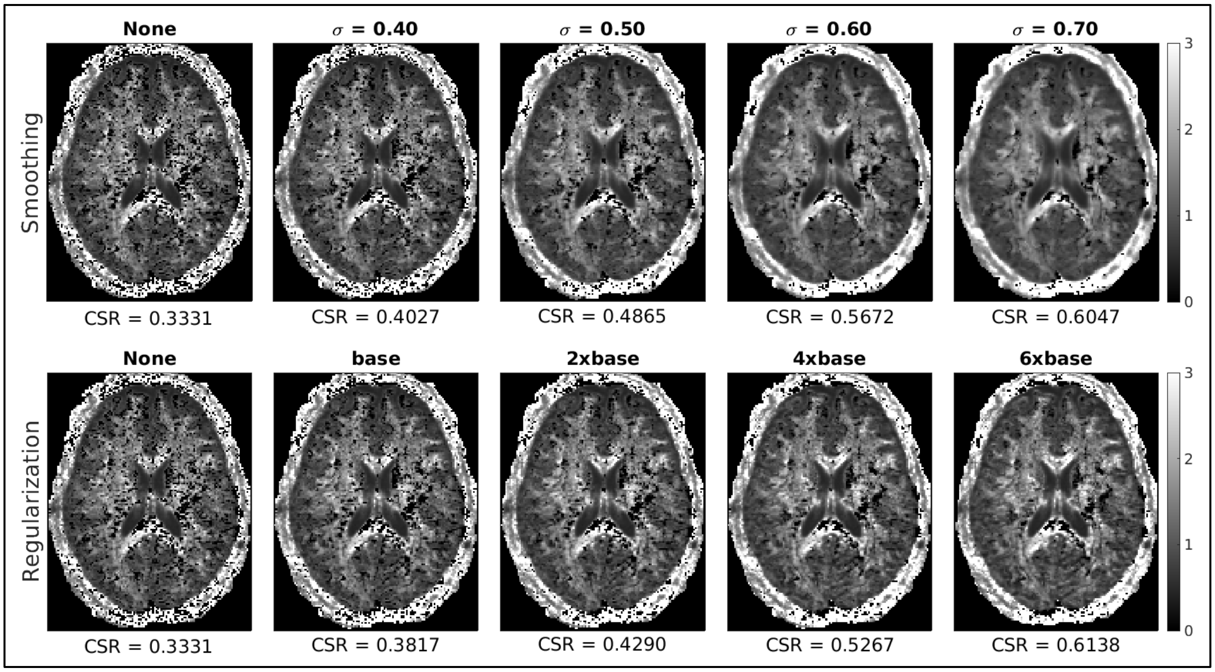

Supplementary Figure 4. *Comparison of $K_{\perp}$ maps with increasing levels of spatial regularization and Gaussian smoothing. σ indicates the standard deviation of the Gaussian kernel used for smoothing on the diffusion-weighted images prior to fitting, in units of voxels. Regularization weighting (base: $\gamma_{DT} = 0.5, \gamma_{DK} = 0.2$) and σ levels were chosen so that in each column there are approximately equal numbers of noisy voxels in each column. Contrast-to-noise ratio (CSR) was calculated using Eq. 13 for each map.*
